# In-vitro model to mimic T cell subset change in human PDAC organoid co-culture

**DOI:** 10.1007/s00432-023-05100-7

**Published:** 2023-07-20

**Authors:** M. Knoblauch, T. Ma, I. Beirith, D. Koch, F. Hofmann, K. Heinrich, U. Aghamaliev, S. Sirtl, C. B. Westphalen, H. Nieß, M. Reichert, M. K. Angele, I. Regel, A. V. Bazhin, J. Werner, M. Ilmer, Bernhard W. Renz

**Affiliations:** 1 Department of General, Visceral, and Transplantation Surgery, LMU Klinikum, Marchioninistr. 15, 81377 Munich, Germany; 2 German Cancer Consortium (DKTK), German Cancer Research Centre (DKFZ), Heidelberg, Germany; 3 Department of Medicine II, University Hospital, Ludwig Maximilian University of Munich, Bavarian Centre for Cancer Research (Bayerisches Zentrum Für Krebsforschung), Munich, Germany; 4 Department of Hematology/Oncology and Comprehensive Cancer Center Munich, LMU University Hospital Munich, Ludwig-Maximilians-University Munich, Munich, Germany; 5 Department of Internal Medicine II, Klinikum Rechts der Isar, Technische Universität München, Munich, Germany; 6 Translational Pancreatic Cancer Research Center, Klinik und Poliklinik für Innere Medizin II, Klinikum rechts der Isar, Technical University of Munich, Munich, Germany; 7 Center for Functional Protein Assemblies (CPA), Technical University of Munich, Garching, Germany; 8 Center for Organoid Systems (COS), Technical University of Munich, Garching, Germany; 9 Munich Institute of Biomedical Engineering (MIBE), Technical University of Munich, Garching, Germany

**Keywords:** Pancreatic cancer, Organoids, Tumor-immune interaction, Pancreatic cancer organoids

## Abstract

**Purpose:**

Immunotherapies have largely failed as treatment options for pancreatic ductal adenocarcinoma (PDAC). In this field, clinical translational studies into personalized treatment are of fundamental importance. In our study, we model tumor-cell immune-cell interactions in a co-culture of primary human PDAC organoids and matched peripheral blood mononuclear cells (PBMCs).

**Methods:**

Using flow cytometry, we evaluated changes in T cell subtypes upon co-culture of patient-derived PDAC organoids and matched PBMCs.

**Results:**

After co-culturing PDAC organoids with PBMCs, we observed changes in CD4^+^, CD8^+^ and Treg cell populations. We observed favorable clinical outcome in patients whose PBMCs reacted to the co-culture with organoids.

**Conclusion:**

This experimental model allows to investigate interactions between patient derived PDAC organoids and their PBMCs. This co-culture system could serve as a preclinical platform to guide personalized therapeutic strategies in the future.

**Supplementary Information:**

The online version contains supplementary material available at 10.1007/s00432-023-05100-7.

## Introduction

Pancreatic ductal adenocarcinoma (PDAC) still carries a dismal prognosis. Because of immune evasion and a generally immunosuppressive tumor microenvironment, PDAC is considered an immunological desert and immunotherapies have largely failed in this disease. Immune evasion is a limiting factor for PDAC progression and metastasis and is instrumental for the overall sobering prognosis (Mundry et al. 2020).

Tumor immunotherapy plays an important role in modern cancer treatment. In this regard efforts in modulating the immune system as a therapeutic strategy are broad (Balachandran et al. 2019; Schizas et al. 2020). These treatment approaches have shown promising results in some cancers, benefits in PDAC are still limited due to an extensive immunosuppression within the tumor microenvironment (Ho et al. 2020; Wiehagen et al. 2017). The immunosuppressive environment in PDAC can lead to a manipulation of tumor cells to evade immune surveillance and is one of the most important factor reasoning the failure of immunotherapy (Banerjee et al. 2018).

Considering the growing incidence of PDAC, translational studies driving our understanding of pancreatic cancer and precision oncology are urgently needed (Sung et al. 2021). A major breakthrough in the modeling of pancreatic cancer was the advent of the organoid technology (Reichert et al. 2013; Renz et al. 2018a, b).

Up to now, organoid models have been favourably established for several cancer entities (Broutier et al. 2017; Sachs et al. 2018; Seino et al. 2018; Vlachogiannis et al. 2018; Weeber et al. 2015). These results underline the importance of translational studies. Co-culture models using Peripheral Blood Mononuclear Cell (PBMCs) are applied to form a great possibility to model the disease and its tumor microenvironment (Cattaneo et al. 2020).

Here, we aimed to characterize changes in certain T cell subtypes from PBMCs in a co-culture model with primary human PDAC organoids.

## Materials and methods

Materials and Methods were in part published in the thesis of Tianmiao Ma (Ma 2022).

### Material

See Key Resources.

### Methods

#### PBMCs isolation from blood samples

Pancreatic tumor specimens and PBMCs were obtained from patients undergoing surgical resection at the Surgical Department of the Ludwig-Maximilians-University (LMU) Hospital from 2020 to 2021 following approval by the Ethics Committee of LMU (#02512). Ten healthy people and 4 PDAC patients were included for blood donation and 4 matching PDAC tissue samples were obtained. The blood collection and processing of all the patients were taken by professionals blinded to the information of the patients in strict accordance with local safety regulations before surgery. Institutional review board approval was obtained.

### PBMC isolation

20 mL of fresh blood were collected in a 50 mL Tube (Falcon, Corning, Mexico) for PBMC isolation diluted in PBS in a proportion of 1:1. 20 mL Biocholl (Biochrom, Berlin, Germany) was used accordingly as a control. The Biocoll was carefully overlaid by blood/PBS mixture without destroying its surface. The different components of peripheral blood were separated into different layers after centrifugation at 20 min/1200rcf/room temperature (RT). Then the intermediate phase (PBMCs) was carefully transferred to a new falcon tube and washed with PBS in proportion 1:4. PBMCs were resuspended in the washing buffer and centrifuged at 10 min/300rcf/RT. The supernatant discarded was followed by centrifugation at 10 min/200rcf/RT, and the PBMC pellets were thoroughly washed again with 20 ml of PBS. The falcon tubes contained with PBMCs were then centrifuged at 10 min/200rcf/RT, and the supernatant was rejected after that. Finally, the loose pellets of PBMCs through adequate vortexing were dissolved in RPMI 1640 medium (RPMI Gibco, Thermo Fisher Scientific, Massachusetts, USA), supplemented with 10% fetalbovine serum (FBS) and 1% penicillin–streptomycin (P/S).

### Organoid culture

Organoid culture was performed according to previous described protocol (Dijkstra et al. 2018). Briefly, fresh tissue derived from surgical resection was placed in 50 ml tube (Falcon, Corning, Mexico) with 10 ml of PBS (PAN BioTECH, Germany) on ice. The tissue was transferred to a 10 cm Petri dish, (Thermo Fisher Scientific, US) minced into small fragments using a scalpel (Thermo Fisher Scientific, US), and added to a GentleMACS tube with 10 ml of prepared digestion buffer. This tube was then fixed and incubated for 2 h at RT with gentle shaking (the program was pre-set in the software). Subsequently, this sample was filtered through the cell strainer (100 μm) to a new falcon tube, added with the required volume of ice-cold PBS + 0.1% BSA to 15 ml, and centrifuged at 5 min/1000 rpm/4 °C. After discarding the supernatant, the pellets were washed with 3 ml of ACK buffer (Gibco Life Technologies, Germany) and incubated at RT for at least 3 min until the red blood cells were invisible, then washed with 5 ml of ice-cold PBS + 0.1%BSA to stop lysis and centrifuged at 5 min/1000 rpm/4 °C before the supernatant was carefully discarded. 3 ml of TrypLE (Gibco 12,563–011, Thermo Fisher Scientific) was added to dissociate the cell clusters into single ones and incubated for 5 min in a 37 °C water bath. Later, the sample was washed with 5 ml of ice-cold PBS + 0.1% BSA and centrifuged at 5 min/1000 rpm/4 °C again to obtain qualified cells for culture. The cell pellets collected after the supernatant discarded were re-suspended in matrigel (Corning, New York, USA) (50 μl/well) and incubated on ice for 5 min. The Matrigel-cell-suspension was slowly transferred into 24-well plates (Thermo Fisher Scientific, US) to form a 3D dome-like structure ensuring air bubble free plating. The cells were incubated for 10-15 min for gel solidification. Each matrigel dome was covered by 500 μl of complete medium prewarmed to 37 °C for long-time culture in the incubator. The medium was refreshed every three days during organoid culture.

### Organoid passaging

Organoids were split every 5–10 days, in a ratio of 1:2. The matrigel-organoid mixture was collected in a new falcon tube until fully washed with cold cell recovery solution and cold PBS (10 ml in total), respectively. The cell pellets were acquired after centrifugation at 5 min/1000 rpm/4 °C and the supernatant was discarded. The desired amount of matrigel (50 μl/well) was added to the dissociated cells and mixed completely on ice. 50 μl of the new matrigel-organoid mixture was pipetted into a 24-well plate, each of them was replenished with 500 μl of pre-warmed complete medium. The plate was then returned to the incubator for further culturing.

### Culture PBMCs with conditioned medium derived from human pancreatic cancer cell lines

Human pancreatic cancer cell lines (Panc1 and Miapaca2) were originally received from American Type Culture Collection (ATCC). Cells were checked quarterly for mycoplasma contamination using the MycoAlert Mycoplasma Detection Kit (Lonza) and authenticated. These cells were separately cultivated in a T-75 flask (Thermo Fisher Scientific, US) with 15 ml of previous described medium, detached with 5 ml 0.025% Trypsin/EDTA solution, and passaged every 3–4 days depending on cell growth. The conditioned medium samples from the supernatant of Panc1 and Miapaca2 after culturing for 72 h were gathered in a new falcon tube and filtered through a sterile 0.20 μm filter to remove cell debris, then frozen at -20 °C, respectively. The PBMCs were cultured with 50% of previous described medium and 50% of Panc1/Miapaca2-derived CM, as an experimental (EXP) group. 48 h later, PBMCs were collected separately and stained for FACS analysis.

### Co-cultured PBMCs with human pancreatic cancer cell lines

Co-culture was performed with modifications as previously described (Dijkstra et al. 2018). Briefly, Panc1/Miapaca2 cells were seeded in a 6-well plate one day before being co-cultured with PBMCs. The next day, one or two vials of frozen PBMCs were thawed in a 37 °C water bath, transferred to a falcon tube, and washed with 9 ml of PBS. After centrifuging at 5 min/500 rcf/RT and discarding the supernatant, the PBMCs were resuspended in the previous described culture medium. The same amount of prepared PBMCs were added to each well at a ratio of 25:1 with Panc1/Miapaca2 cells. Waiting for 48 h, the PBMCs in the control (CON) group and the mixture of PBMCs and Panc1/Miapaca2 in the bottom 3 wells EXP group were collected respectively and then stained for flow cytometry.

### Co-cultured PBMCs with organoids from PDAC patients

The co-culture of PBMCs and organoids was performed with modifications as previously described (Dijkstra et al. 2018). The PBMCs were resuspended in the organoid complete medium, added to the certain wells with organoids-matrigel mixtures, and cultured in the incubator for 48 h, which served as an EXP group. In the CON group, the same numbers of PBMCs were cultured alone in the same medium and matrigel for 48 h. The staining of PBMCs for flow cytometry was performed after cell collecting in the control group and co-culture group, respectively. Autologous co-cultures were established with PDAC patient-derived organoid lines and their matched PBMCs.

### Flow Cytometry

The PBMCs were re-suspended in the desired medium at 1 × 10^6^ cells/ml and transferred to the FACS tubes (200 μl/tube). The certain tubes in both CON and EXP group were added with 1 μl of each antibody, vortexed, and incubated in a dark chamber for 15-30 min/RT. The samples were washed with 2 ml of FACS buffer (PBS + 2%NaN_3_ + 5%BSA) completely and centrifuged at 5 min/500 rcf/RT. After discarding the supernatant and adding another 500 μl of FACS buffer in each tube, the samples were measured by BD LSRFortessaTM Cell Analyzer and according to the gating strategy which you can see in Supplementary Material. The data were recorded and downloaded from BD FACS Diva 8.0.1 software and imported to Flowjo10 for further analysis. The gating strategies of Memory T cells and Tregs on the FACS plots were shown in Suppl. Material Figure S2 A, B.

### Statistical Analysis

The Student’s t-test to evaluate the differences between control and experimental groups or one/two-way analysis of variance (ANOVA) for multiple group comparisons were applied in all statistical analyses by GraphPad Prism 9 (Graphstad, US) based on the population of immune cells. P < 0.05 was considered to be statistically significant.

## Results

### Frequency of effector, effector memory and central memory T cells was higher in PBMCs of PDAC patients compared to healthy donors

First, we aimed to determine the baseline for T cell subsets in PBMCs obtained from healthy donors (HD) and PDAC patients (PDAC-D) as a prerequisite for the following experiments. The analysis revealed that the frequency of effector (Teff), memory (Tem) and central memory T cells (Tcm) of CD4^+^ cells was higher in PBMCs from PDAC-D than from HD (p < 0.001, Supplementary Fig. 1A). The frequencies of Tnaiv cells for both CD4^+^ and CD8^+^ were lower in PDAC-D when compared to HD (p < 0.001, Supplementary Fig. 1B). In a similar manner, frequency of CD8^+^ Teff, Tem and Tcm cells in PBMC (PDAC-D) group was found to be significantly higher than those in PBMC (HD) group (p < 0.05, Supplementary Fig. 1B). Frequency of regulatory T cells (Tregs) in PBMCs of PDAC-D was also higher than in healthy PBMCs (data not shown, p < 0.05).

As expected, we found that the constellation of immune cell subsets in PDAC patients differ from those in healthy patients.

### Direct cell–cell contact is necessary for immune cells to effect immune response

Next, we investigated effects of conditioned medium (Topalian et al.) containing soluble factors (i.e. cytokines, chemokines, etc.) to induce changes in T cell subsets during cultivation without direct cell–cell contact.

**Fig. 1:**
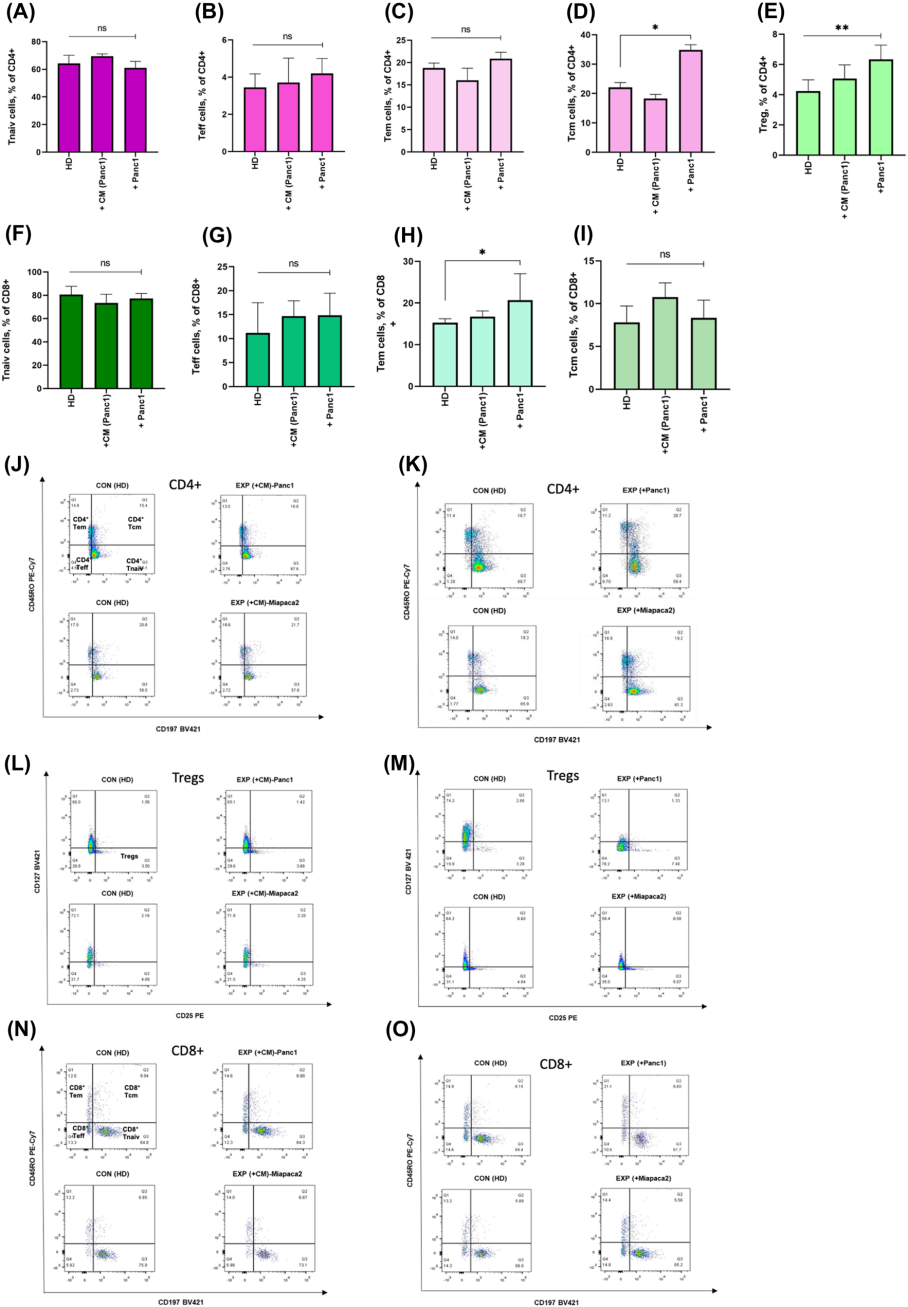
Direct immune-tumor cell contact is necessary to modulate immune response. Co-Culturing PBMCs (HD) with CM (Panc1/MiaPaca2), and Panc1 cells. **A** The frequency of CD4^+^ Tnaiv cells showed no statistically significant differences in each co-culture group. **B** The frequency of CD4^+^ Teff cells showed no statistically significant differences in each co-culture group. **C** The frequency of CD4^+^ Tem cells showed no statistically significant differences in each co-culture group. **D** The frequency of CD4^+^ Tcm cells was higher in the EXP (+ Panc1) group than that in the CON (HD) group (**P < 0.01, *P < 0.05, and *P < 0.05). **E** The Treg frequency in the EXP (+ Panc1) group was higher than that in the CON (HD) group (**P < 0.001). **F** The frequency of CD8^+^ Tnaiv cells showed no statistically significant differences in each co-culture group. **G** The frequency of CD8^+^ Teff cells showed no statistically significant differences in each co-culture group. **H** CD8^+^ Tem population was significantly higher in the EXP (+ Panc1) group than that in the CON (HD) group (**P < 0.01, *P < 0.05, and *P < 0.05). **I** The population of CD8^+^ Tcm cells showed no statistically significant differences in each co-culture group. **J** FACS plot of Memory CD4^+^ T cell subsets in the CON (HD) and EXP (+ CM Panc1/MiaPaca2) groups. **K** FACS plot of Memory CD4^+^ T cell subsets in the CON (HD) and EXP (+ Panc1/MiaPaca2) groups. **L** FACS plot of Tregs in the CON (HD) and EXP (+ CM Panc1/MiaPaca2) groups. **M** FACS plot of Tregs in the CON (HD) and EXP (+ Panc1/MiaPaca2) groups. **N** FACS plot of different Memory CD8^+^ T cell subsets in the CON (HD) and EXP (+ CM Panc1/MiaPaca2) groups. **O** FACS plot of different Memory CD8^+^ T cell subsets in the CON (HD) and EXP (+ Panc1/MiaPaca2) groups

Therefore, we cultured PBMCs from HD in CM from PDAC cells (Panc1/MiaPaca2). We did not observe significant changes in different subsets of CD4^+^ (CM-Panc1; Fig. 1A–E column 1–2, CM-MiaPaca2; Supplementary Fig. 1C) and CD8^+^ Tnaiv, Teff, Tem, Tcm and Treg compared to PBMCs from HD cultured alone (Fig. 1F–I, column 1–2). Taken together, cultivation of PBMCs from healthy donors in conditioned medium of PDAC cells did not induce changes in the T cell subsets of PBMCs.

Next, we co-cultivated PBMCs (HD) with Panc1/MiaPaca2 cells. In these experiments we observed changes in CD4^+^ Tcm and CD8^+^ cells. Co-culturing PBMCs with Panc1 cells (EXP-Panc1-HD) led to an increase in CD4^+^ Tcm cells when compared to controls (CON-HD) (*p* = 0.04, Fig. 1 D, column 3).

Frequency of Treg in EXP-Panc1-HD also increased after co-cultivation with cells from both cell lines (*p* = 0.04, Fig. 1 E, column 3). CD8^+^ Tem frequency in the Panc1-HD group was significantly higher than in the CON-HD group (*p* < 0.05, Fig. 1 H, column 3). Panc1 cells were more potent to induce effects on immune cell differentiation than MiaPaca2 cells.

Direct cell–cell contact led to changes in T cell subtype frequencies and as expected, co-cultivation of PBMCs with PDAC cells led to a higher amount of memory and regulatory T cells.

### Co-culture of primary human PDAC organoids and PBMCs from HD effected heterogenous phenotype change in T cell subsets

**Fig. 2:**
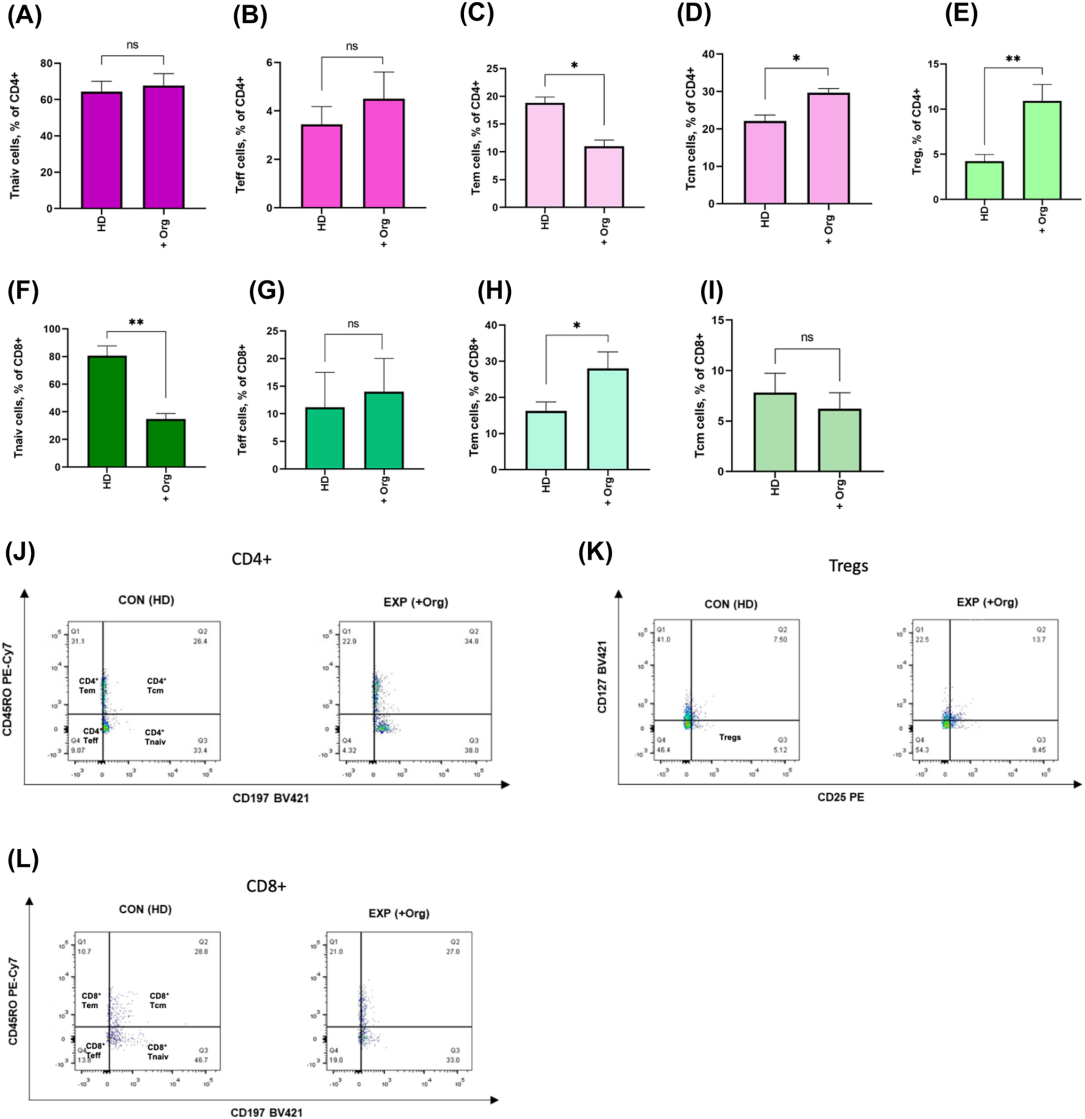
Co-culture of primary human PDAC organoids and PBMCs from HD effect heterogenous phenotype change in T cell subsets. **A** The frequency of CD4^+^ Tnaiv cells showed no statistically significant differences in co-culture group. **B** CD4^+^ Teff cells were larger in the EXP (+ Panc1) group than that in the CON group but not significantly. **C** The frequency of CD4^+^ Tem cells was lower in the EXP (+ Org) group than that in the CON (HD) group (**P < 0.01, *P < 0.05, and *P < 0.05). **D** The frequency of CD4^+^ Tcm cells was higher in the EXP (+ Org) group than that in the CON (HD) group (**P < 0.01, *P < 0.05, and *P < 0.05). **E** The Treg frequency in the EXP (+ Org) group was higher than that in the CON (HD) group (**P < 0.001). **F** CD8^+^ Tnaiv frequency was lower in EX*P* (+ Org) group than in CON (HD) group (**P < 0.01, *P < 0.05, and *P < 0.05). **G** The frequency of CD8^+^ Teff cells showed no statistically significant differences in co-culture group. **H** CD8^+^ Tem frequency was significantly higher in the EXP (+ Org) group than that in the CON (HD) group (**P < 0.01, *P < 0.05, and *P < 0.05). **I** The frequency of CD8^+^ Tcm cells showed no statistically significant differences in co-culture group. **J** FACS plot of different Memory CD4^+^ T cell subsets. **K** FACS plot of Tregs. **L** FACS plot of different Memory CD8^+^ T cell subsets

For the following experiments we co-cultured primary human PDAC organoids directly with PBMCs from healthy donors. In these experiments, we did not observe differences in the frequency of CD4^+^ Teff and Tnaiv between those two groups (*p* > 0.05, Fig. 2A, B).

Co-cultivation of PBMCs from HD with PDAC organoids (PDAC-Org-HD) led to a decrease in the amount of Tem cells, while the amount of Tcm cells increased (*p* = 0.04, Fig. 1D). Frequency of CD4^+^ Tem in PBMCs from HD alone and in PDAC-Org-HD was  also significantly lower (*p* = 0.03, Fig. 2C). In addition, the amount of CD4^+^ Treg increased in PDAC-Org-HD compared to PBMC-HD (*p* < 0.001, Fig. 1E). Concerning CD8^+^ Tcells, co-cultivation of PBMCs with PDAC organoids led to a decrease in the amount of Tnaiv cells as well as an increase in Tem cells in PDAC-Org-HD (Fig. 2F, H). CD8^+^ Teff and Tcm cells were not affected in PDAC-Org-HD (Fig. 2G, I).

Direct contact between immune cell and tumor organoid led to higher frequency of CD8^+^ Tem, CD4^+^ Tcm and Treg cells. A lower frequency of CD4^+^ Tem and CD8^+^ Teff cells was obtained in co-culture of PDAC organoids and PBMCs from healthy donors.

### Co-culture of PBMCs from PDAC patients and Panc1 cells caused heterogenous phenotype change in T cell subsets

As described above, we identified differences in subsets of T cells in PBMCs from HD co-cultivated either with cell lines or with organoids. Next, we aimed to investigate the effect of co-cultivation on PBMCs from PDAC-D with pancreatic cancer cells.

**Fig. 3:**
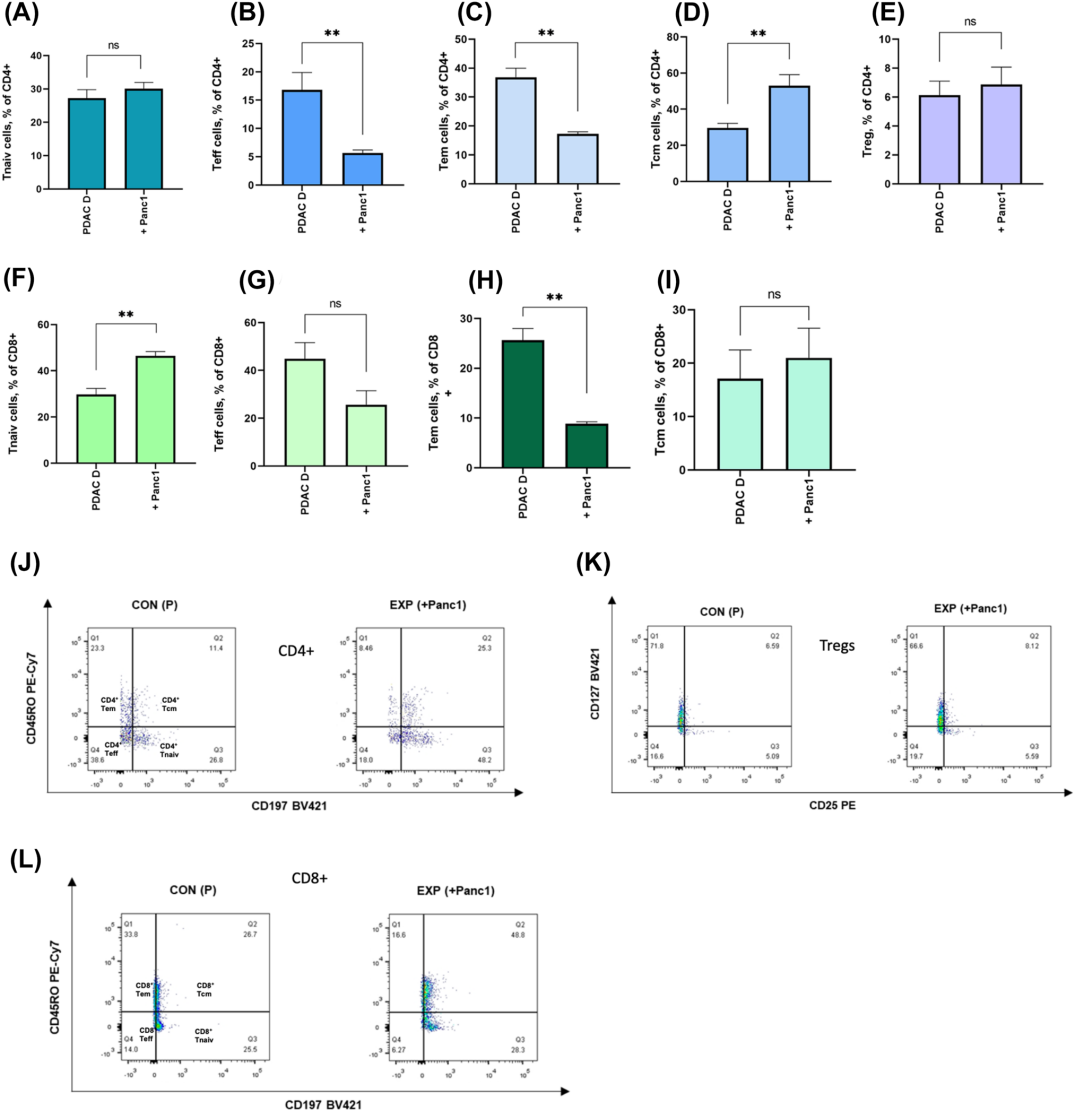
Co-culture of PBMCs from PDAC patients (PDAC-D) and Panc1 cells causes heterogenous phenotype change in T cell subsets. **A** The frequency of CD4^+^ Tnaiv cells showed no statistically significant differences in co-culture group. **B** CD4^+^ Teff cells were lower in the EXP (+ Panc1) group than that in the CON (P) group (**P < 0.01, *P < 0.05, and *P < 0.05). **C** CD4^+^ Tem cells were lower in the EXP (+ Panc1) group than that in the CON (P) group (**P < 0.01, *P < 0.05, and *P < 0.05). **D** The frequency of CD4^+^ Tcm cells was higher in the EXP (+ Panc1) group than in the CON (P) group (**P < 0.01, *P < 0.05, and *P < 0.05). **E** The frequency of CD4^+^ Treg cells showed no statistically significant differences in co-culture group. **F** The frequency of CD8^+^ Tnaiv cells was higher in the EXP (+ Panc1) group (**P < 0.01, *P < 0.05, and *P < 0.05). **G** CD8^+^ Teff cells were lower in the EXP (+ Panc1) group than that in the CON (P) group but not significantly. **H** CD8^+^ Tem cells were lower in the EXP (+ Panc1) group than that in the CON (P) group (**P < 0.01, *P < 0.05, and *P < 0.05). **I** CD8^+^ Tcm cells did not change significantly. **J** FACS plot of different Memory CD4^+^ T cell subsets in the CON (P) and EXP (+ Panc1) groups. **K** FACS plot of Tregs in the CON (PDAC D) and EXP (+ Panc1) groups without significant differences. **L** FACS plot of different Memory CD8^+^ T cell subsets in the CON (P) and EXP (+ Panc1) groups

Therefore, we co-cultured PBMCs from PDAC-D with Panc1 cells. The CD4^+^ Tnaiv frequency in PBMCs (PDAC-D) cultured with Panc1 was not different to the CD4^+^ Tnaiv frequency in PBMCs (PDAC-D) cultured alone (Fig. 3A). CD4^+^ Tem and Teff frequencies in co-culture were both lower than those in PBMCs from PDAC donors alone (p = 0.001 and 0.016, Fig. 3B, C). The frequency of CD4^+^ Tcm cells in PBMCs (PDCA-D) co-cultured with Panc1 cells increased significantly (p = 0.013, Fig. 3D). No differences were seen in the frequency of Treg in PBMCs (PDAC-D) and PDAC-D-Panc1 (Fig. 3E). Frequency of CD8^+^ Tnaiv cells in PDAC-D-Panc1 group was higher than that in PDAC-D (p < 0.01, Fig. 3F). Moreover, there was a decrease in CD8^+^ Teff frequency and an increase in CD8^+^ Tcm frequency in the PDAC-D-Panc1 compared to PDAC-D alone (Fig. 3G, K). However, CD8^+^ Tem cells (PDAC-D-Panc1) were significantly lower in the co-culture group than in the control group (PDAC-D) (*P* = 0.002, Fig. 3H).

Co-culture of Panc1 cells and PBMCs (PDAC-D) resulted in a lower frequency of CD4^+^ and CD8^+^ Tem and Teff cells than those in PBMCs from PDAC donors alone. The frequency of CD4^+^ Tcm cells, CD8^+^ Tnaiv and Tcm cells in PBMCs from PDAC donors co-cultured with Panc1 cells increased significantly.

### Co-culture of matched PBMCs with primary human PDAC organoids displayed heterogenous T cell response

Next, we utilized this co-culture model employing PBMCs and primary PDAC organoids from the same patient to mimic T cell response.

**Fig. 4:**
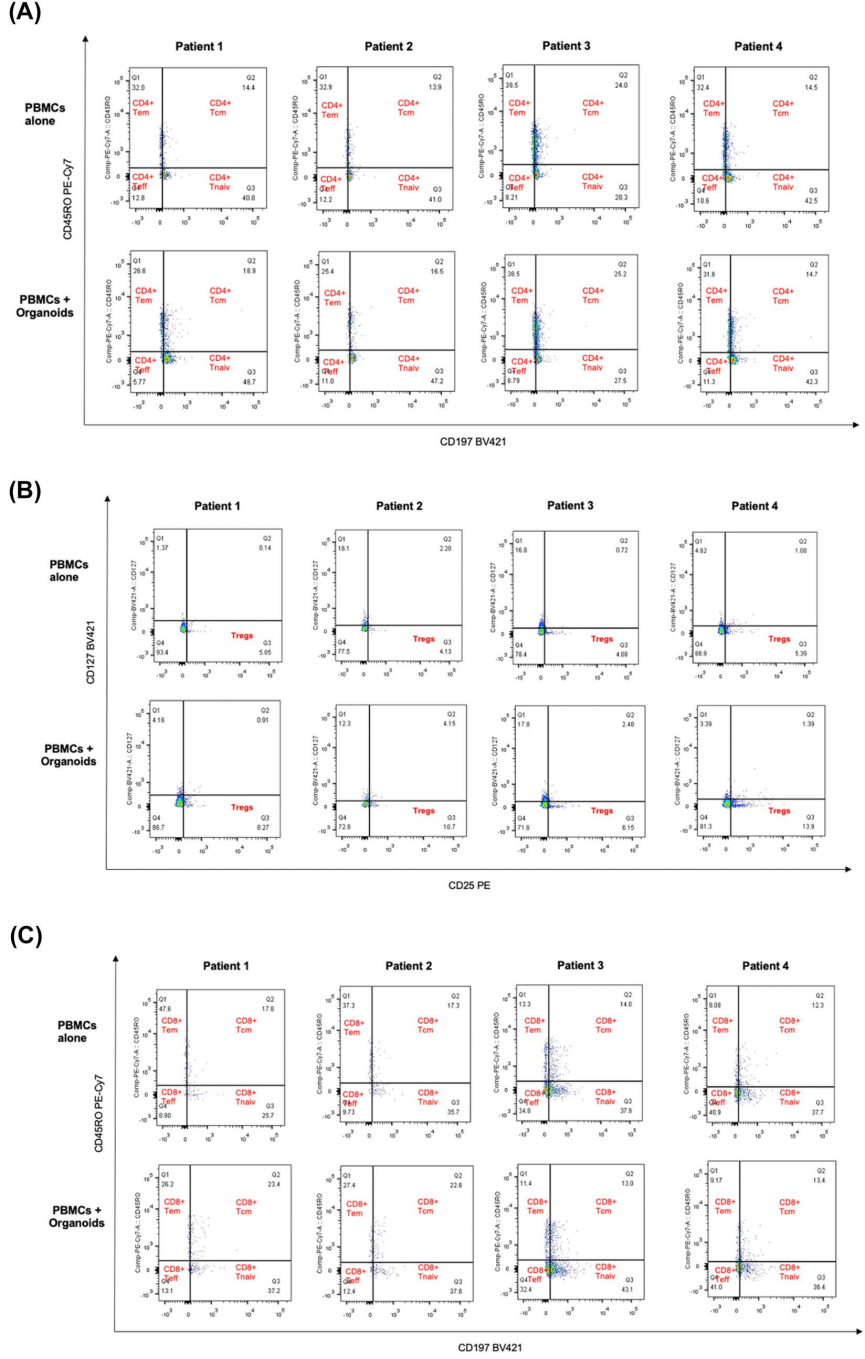
Individual co-cultures of matched PBMCs with primary human PDAC organoids. **A** CD4^+^ Memory T cell subsets showed personal variations between the CON (P) and EXP (+ Org) groups of Patient 1, 2, 3, and 4. **B** Frequency of Tregs was increased in the co-cultures from Patient 1, 2, 3, and 4 compared to the control group. **C** CD8^+^ Memory T cell subsets showed personal variations between the CON (P) and EXP (+ Org) groups of Patient 1, 2, 3, and 4

In patient 1 (P1) CD4^+^ Tem and Teff cell population in PDAC-Org-P1 were lower than in the PBMC-P1 group. The CD4^+^ Tcm and Tnaiv cells in PDAC-Org-P1 were both higher than those in the PBMC-P1 (Fig. 4A). Frequency of CD4^+^ Tem cells decreased in  the PBMC-P2 group and in PDAC-Org-P2 while frequency of CD4^+^ Tcm and CD4^+^ Tnaiv cell population slightly increased. In contrast, CD4^+^ Teff cells showed only minor differences in their frequency between these two groups. However, frequency of Treg cells in PDAC-Org-P2 and PDAC-Org-P3 were higher than in PBMC-P2 and PBMC-P3. We were able to detect similar changes in the CD8^+^ memory T cell subpopulation in PDAC-Org-P1/2 compared to autologous PBMCs (Fig. 4C).

In co-culture of P1 and P2, we could see an increase in CD4^+^ and CD8^+^ Tcm, Tnaiv and Treg cells as well as a decrease in CD4^+^ and CD8^+^ Tem cells. It should be noted that we did not see any differences in memory CD4^+^ and CD8^+^ T cell subtypes in co-culture of P3 and P4 compared with their controls. In contrast, there was a significant increase in frequency of Treg cells in PDAC-Org-P3 and PDAC-Org-P4 (8.15%/13.9%) compared to PBMCs alone (4.08%/5.39%) (Fig. 4B).

**Fig. 5:**
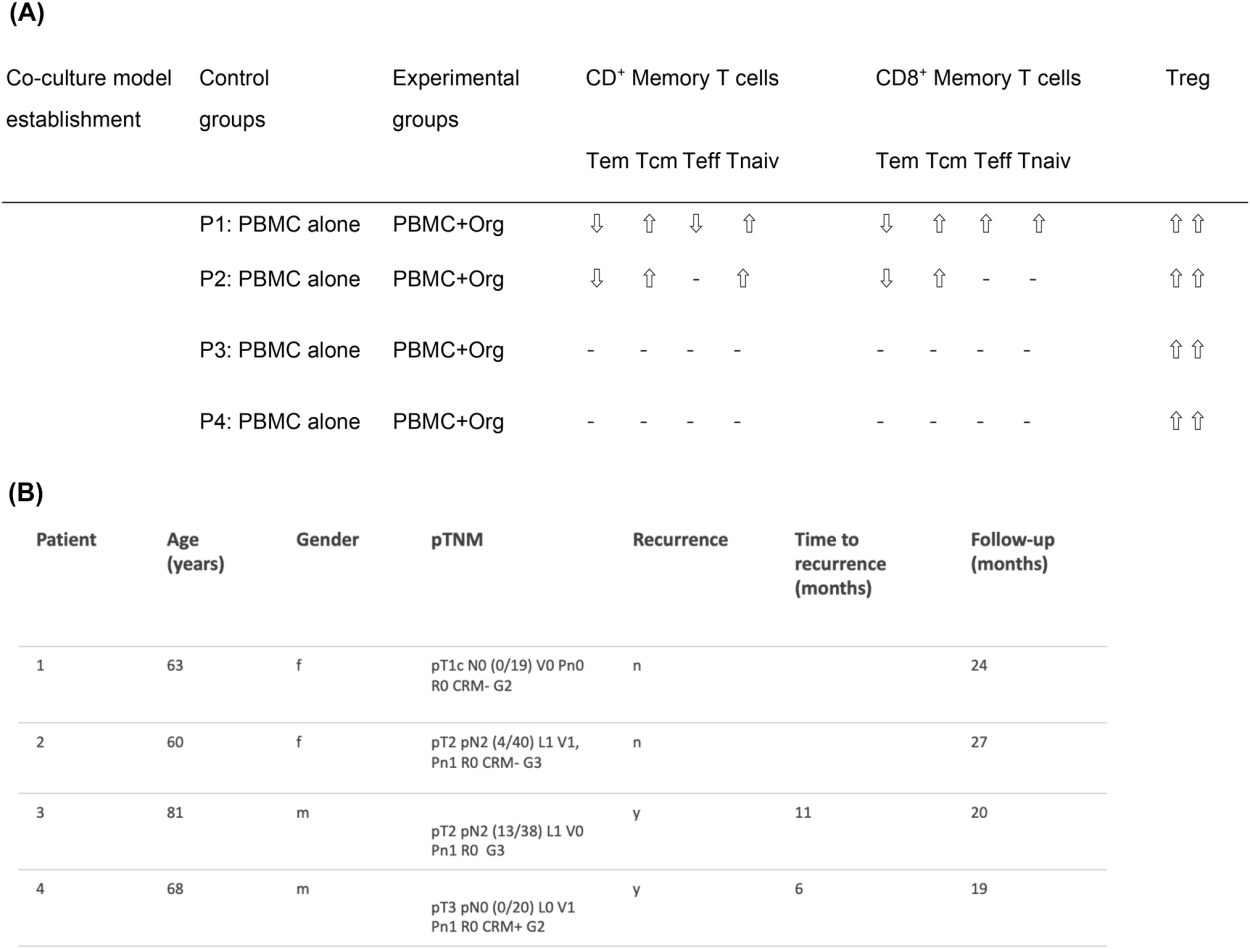
**A** Results of the autologous co-culture model with PBMCs and PDAC organoids. **B:** Patients with T cell subset change in co-culture were younger and show longer recurrence-free survival in our study than those without immune reaction in co-culture

Our results showed an individual response in co-culture of matched PBMCs with primary human PDAC organoids compared to PBMCs cultured alone (Fig. 5A).

### We observed favorable clinical outcome in patients whose PBMCs reacted to the co-culture with organoids.

We can empathize that patients with obvious changes in T cell subset in PBMCs after co-cultivation with autologous PDAC organoids (P1 and P2) were younger and showed longer free survival than those without these changes. Patients without T cell subset change in co-culture had tumor recurrence six and eleven months after surgery during follow-up (Fig. 5B).

## Discussion

We evaluated differences in T cell subsets after co-culture in individual PDAC patients. Co-culture of PBMCs with established PDAC cell lines and CM guided us to optimal conditions for PBMC and PDAC organoid co-culture. CD4^+^ and CD8^+^ T cell differentiation varied in 4 patient co-cultures (EXP-PDAC-D-Org1-4 vs. PDAC-D) and Treg cell frequency was higher in all co-cultures than in their matched controls. This model reflects the heterogeneity of tumor-immune interactions in individual patients. It might serve as a preclinical model helping to guide treatment options of PDAC patients in the future.

Organoid cultures have been studied for individualized tumor response assessment in different cancers (Shi et al. 2020; van de Wetering et al. 2015). Tumor-specific T cell responses for colorectal cancer and non-small lung cancer are already using similar co-culture conditions (Dijkstra et al. 2018). However, this model with patient-derived PDAC organoids is to our knowledge not yet examined. Previous studies have examined such co-culture models partially similar but focused on lymphocyte infiltration (Tsai et al. 2018). Moreover, previous experiments did not directly focus on T cell subsets in PBMC and PDAC organoid co-culture.

Our analysis revealed that immune cell subsets in PDAC patients differ from those in healthy patients. These results are congruent with previous published data in other cancer types (Krijgsman et al. 2019; Lulu et al. 2021). We observed that conditioned medium (CM) of PDAC cells did not effect changes in T cell subsets in PBMCs detected by flow cytometry. Our observation that CM is not sufficient to influence immune cells is consistent with the fact that conditioned medium alone is not able to mimic the immune environment in tumors, especially without interactions between different cell types (Dowling & Clynes 2011).

We found increased frequencies of CD4^+^ Tcm and CD8^+^ Tem cells in PBMCs (HD) co-cultured with Panc1 cells compared to PBMCs from HD cultured alone. An activation of CD8^+^ T cells by tumor antigen presentation is previously described (Holokai et al. 2020). This observation can be explained by a lower activation threshold of memory T cells than naïve T cells (Liu et al. 2020; MacLeod et al. 2010). We also noticed an ascending trend in Treg cell frequency which is complementary to previous studies. Treg cells can secrete immunosuppressive cytokines such as IL-10 and TGF-ß which can increase Treg cell frequency (Oleinika et al. 2013). In case of patients PBMCs cultivated with Panc1 cells, we found individual responses in T cell subset changes. Taken together, even those established cell lines behave differently in terms of immune modulation. Besides, we also found enhanced frequency of Treg in patients’ PBMCs. Similar results were found in other solid tumors (Bates et al. 2006; Griffiths et al. 2007; Hiraoka et al. 2006).

Our results confirmed that the amount of CD4^+^/CD8^+^ Tem, Tcm, and Teff cells was higher in PBMCs from PDAC patients than in PBMCs from HD. The frequency of CD4^+^/CD8^+^ Tnaiv cells was significantly lower in patients’ PBMCs, compared to healthy PBMCs which is congruent to existing literature (Hang et al. 2019; Liu et al. 2015).

Our results show an individual response in co-culture of matched PBMCs with autologous primary human PDAC organoids compared to PBMCs cultured alone. Previous published studies can partially explain our observations. The enhancement of Treg can inhibit anti-tumor immune responses in PDAC and results in dismal disease prognosis (Bouneaud et al. 2005). Our heterogenous results in co-culture highlight the importance to better define the compositions of PDAC stroma in individual patients.

Interestingly, patients whose PBMCs respond to the co-culture with organoids were younger and showed a longer recurrence-free survival in our study compared to those without immune reaction in co-culture. Several studies have suggested that a high frequency of CD4^+^ and CD8^+^ T cells in PDAC microenvironment is associated with a better disease-free survival and/or overall survival (Balachandran et al. 2019; Lohneis et al. 2017; Wang et al. 2017). Larger sample size will be needed to underline our results more precisely.

## Conclusion

In this project, we detected different T cell subset changes in co-cultures of PDAC organoids and matched PBMCs. This model carries great potential to facilitate individual treatment strategies in PDAC patients.

## Key Resources

**Table:** 

Reagents, chemicals, and buffer	
1 × Phosphate-buffered saline (PBS)	PAN BioTECH, Germany
A83-01	Tocris Bioscience, England
ACK Lysing buffer	Gibco Life Technologies, Germany
Advanced DMEM/F-12	Gibco Life Technologies, Germany
B27 supplement	Gibco Life Technologies, Germany
Biocoll	Biocell Technology, Germany
Bovine serum albumin (BSA)	Biomol, Germany
CASY Ton	OMNI Life Science, German
Collagenase Type II	Thermofischer, US
Dimethylsulfoxide (DMSO)	ROTH, Germany
EGF recombinant human protein	Gibco Life Technologies, Germany
Fetal Bovine Serum (FBS)	Thermo Fisher Scientific, US
FGF-10 recombinant human protein	Peprotech, Germany
Fixation buffer	Invivogen, US
GlutaMAX supplement	Gibco Life Technologies, Germany
BD Golgistop	BD Biosciences, US
Matrigel (growth factor reduced)	Sigma Aldrich, US
Penicillin/Streptomycin (P/S)	PAN BioTECH, Germany
Perm buffer	Invivogen, US
Primocin	Invivogen, US
Protein Transport Inhibitor	BD Golgistop, US
Recombinant Human R-Spondin 1 protein	R&D systems, US
Recovery cell culture freezing medium	Thermo Fisher Scientific, US
ROCK inhibitor (Y-27632)	Sigma Aldrich, US
RPMI1640	Gibco Life Technologies, Germany
TrypLE express enzyme (1X)	Gibco Life Technologies, Germany
Wnt3a recombinant human protein	R&D systems, US
Antibodies	
CD45 BUV650	BD Bioscience, US
CD3 PerCP-Cy5.5	Biolegend, US
CD4 BUV395	BD Bioscience, US
CD8 APC-H7	BD Bioscience, US
CD25 PE	BD Bioscience, US
CD127 BV421	BD Bioscience, US
CD197 BV421	BD Bioscience, US
CD45RO PE-Cy7	BD Bioscience, US

### Supplementary Information

Below is the link to the electronic supplementary material.

Supplementary file1 (DOCX 1035 kb)

## Data Availability

Not applicable.

## Abbreviations

BSA: Bovine serum albumin
CON: Control
DCs: Dendritic cells
DMEM: Dulbecco's modified eagle medium
DMSO: Dimethyl sulfoxide
EXP: Experiment
FACS: Fluorescence activated cell sorter
FBS: Fetal bovine serum
HD: Healthy donors
IFN-γ: Interferon-gamma
IL: Interleukin
Org: Organoid
P: Patient
P/S: Penicillin/streptomycin
PBMC: Peripheral blood mononuclear cell
PBS: Phosphate-buffered saline
PDAC: Pancreatic ductal adenocarcinoma
Rcf: Relative centrifugal force
rpm: Revolutions per minute
RPMI 1640: Roswell Park Memorial Institute 1640
RT: Room temperature
Tcm cells: Central memory T cells
Teff cells: Effector T cells
Tem cells: Effector memory T cells
TGF-β: Transforming growth factor beta
Tnaiv cells: Naïve T cells
Tregs: Regulatory T cells
