# Supplementary material for: In-vitro model to mimic T cell subset change in human PDAC organoid co-culture
Source: J Cancer Res Clin Oncol. 2023 Jul 20;149(14):13051–64. doi: 10.1007/s00432-023-05100-7 (PMC10587248; doi:10.1007/s00432-023-05100-7)
Supplement: Supplementary file 1 — Supplementary file1 (DOCX 1035 kb) [file 432_2023_5100_MOESM1_ESM.docx]

**Supplementary Material**

**
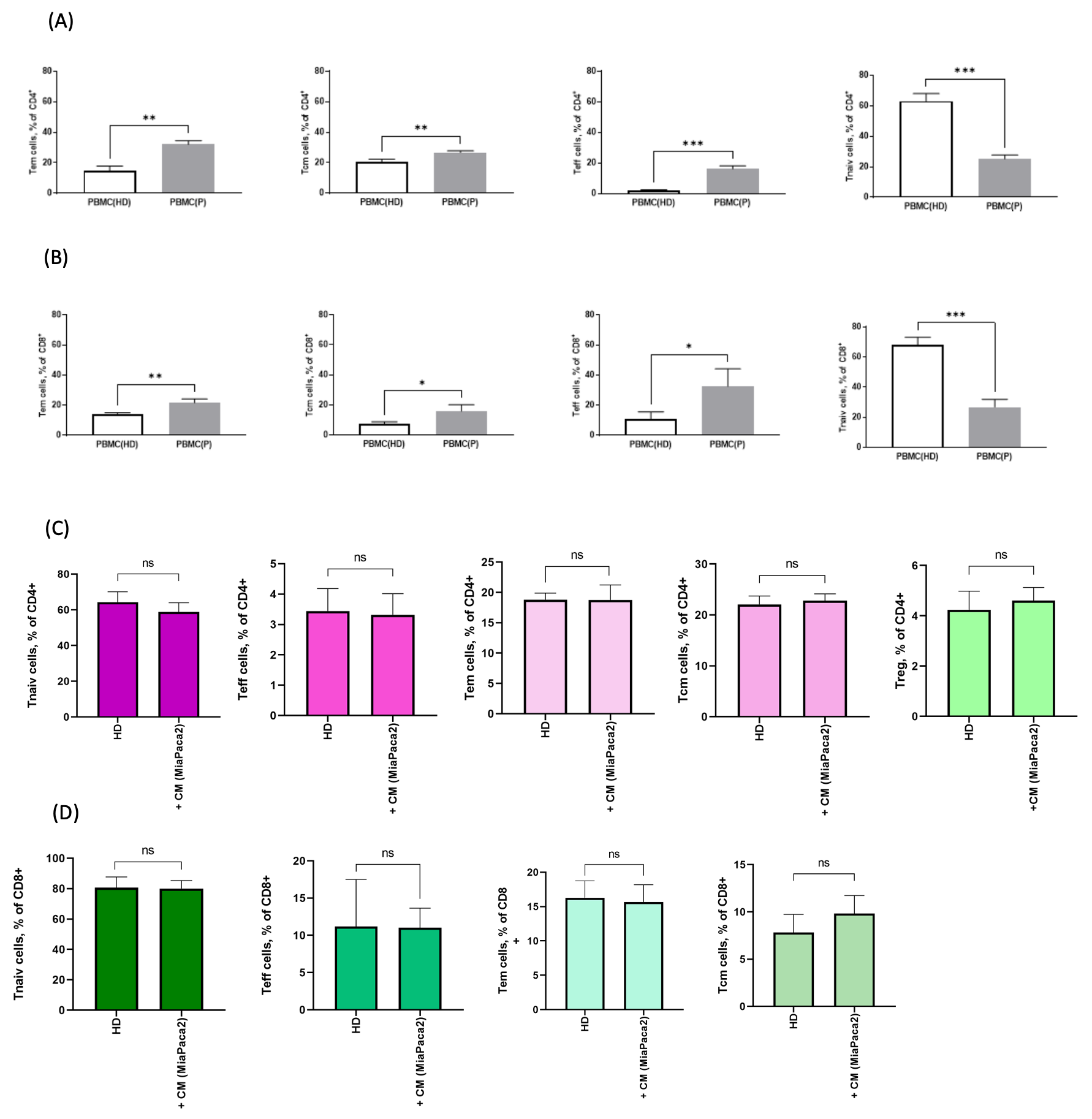
**

**Figure 1:**

1. Frequency of CD4^+^ effector, effector memory and central memory T cells (Teff, Tem and Tcm) is higher in PBMCs of PDAC patients (PDAC-D) compared to healthy donors (HD).
2. Frequency of CD8^+^ effector, effector memory and central memory T cells (Teff, Tem and Tcm) is higher in PBMCs of PDAC patients (PDAC-D) compared to healthy donors (HD).
3. Co-Culturing PBMCs (HD) with CM (MiaPaca2), and MiaPaca2 cells.

**
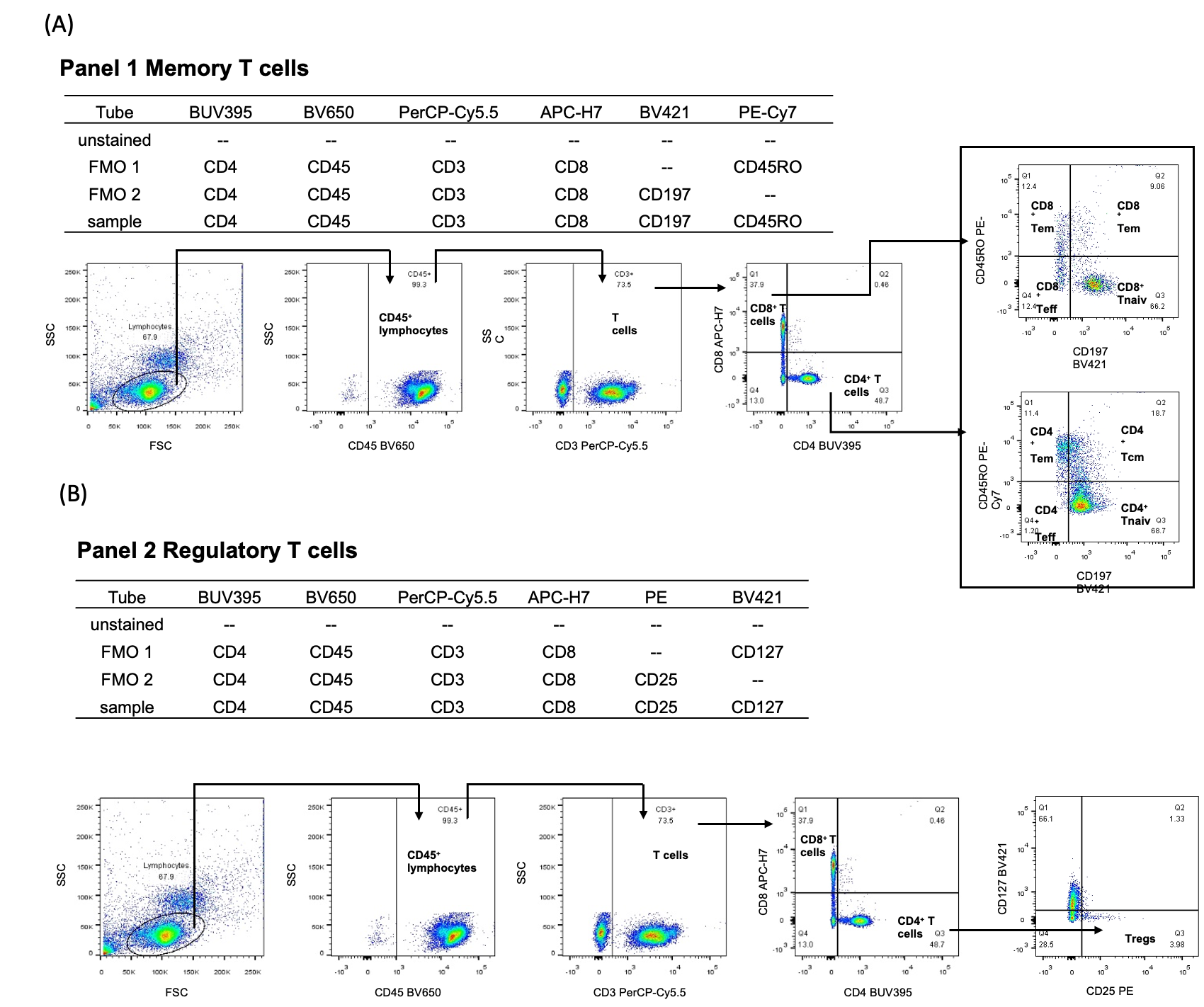
**

**Figure 2:**

1. Gating strategy of Memory T cells in FACS plot.
2. Gating strategy of Tregs in FACS plot.
